# Milk and Contagion

**Published:** 1884-02

**Authors:** 


### MILK AND CONTAGION.

An experiment made some time since by Bollinger deserves more serious consideration than it has received. He took half of a litter of pigs from a healthy sow and fed them with the milk of a scrofulous cow, the diseased condition of which was afterward proved by post-mortem examination. Two pigs were killed at regular intervals, one of those fed on the cow's milk, the other on the milk of the sow. All the latter were found to be healthy, while all of those fed on the scrofulous milk were more or less affected with tuberculosis. Now that stall feeding so largely prevails, especially in and about large towns, the animals thus deprived of fresh air and natural exercise are liable to very human-like diseases. Consumptive cows are common enough, and there is good reason to conclude that human beings are as susceptible as pigs to scrofulous contagion. This is especially the case with delicate children. In all cases where commercial milk from unknown cows is used it should be boiled, in order to destroy the germs (bacteria, probably) of disease, whether they be germs derived from the parent cow or foreign germs introduced by subsequent contact of the milk with impure air or impure hands or vessels. We lose a little by such boiling, the "scum" which floats on the top of boiling milk being the albumen which is coagulated by the heat, and usually, though not necessarily, wasted. Condensed milk is free from the risk of contagion, having been reduced in bulk by boiling, and raised to the boiling point of syrup, which is considerably higher than that of pure water or ordinary milk.

---

*Dr. E. H. Gibbs, Editor Hall's Journal of Health,* 21 *Clinton place* (*Eighth street*), *New York:*

DEAR SIR: We take the liberty of inclosing, for insertion in your valuable journal, a letter received from a medical gentleman, which explains itself:

"Having been much interested in observing both the therapeutic and hygienic results following the employment of Parke, Davis & Co.'s Desiccated Bullock's Blood (*sanguino bovino exsiccatus*), my attention was particularly arrested by a valuable editorial article on 'Nutriment' in HALL'S JOURNAL OF HEALTH for November last, in which reference is made to the use of this preparation per rectum. I can fully indorse all the favorable mention therein made of it, but am obliged to differ with the writer in his instructions concerniug the method of preparing it for rectal use. In the absence of instructions on that particular point it is fair to presume that he would place the blood-powder into the hollow suppository without previous preparation. I should not expect for apparent reasons to derive the best effects from this method. My method, which I can indorse on the basis of large experience, consists in reducing the powder to a thick paste by the addition of water. In preparing this paste it is important that the water be not poured on the scales, but *that the scales be thrown on the water.* Cold water should be employed and the mixture gently stirred with a glass rod or spatula until the scales break down and become incorporated. By pouring the water on the scales agglomeration of the latter can scarcely be avoided and great difficulty is experienced in making the paste. The paste thus prepared and placed in the hollow suppositories is usually quite perfectly absorbed. In cases in which absorption appears to be imperfect it can be very much facilitated by incorporating, say from ten to fifteen grains of saccharated pepsin, in each dose. Of course each dose the blood must be prepared as above as it is required for use. In the presence of moisture decomposition would soon ensue."

"A SUBSCRIBER."

